# External validation of a deep-learning model to predict severe acute kidney injury based on urine output changes in critically ill patients

**DOI:** 10.1007/s40620-022-01335-8

**Published:** 2022-05-12

**Authors:** Francesca Alfieri, Andrea Ancona, Giovanni Tripepi, Vincenzo Randazzo, Annunziata Paviglianiti, Eros Pasero, Luigi Vecchi, Cristina Politi, Valentina Cauda, Riccardo Maria Fagugli

**Affiliations:** 1 Department of Applied Science and Technology, Politecnico di Torino, C.so Duca degli Abruzzi 24, 10129 Turin, Italy; 2 Department of Electronics and Telecommunications, Politecnico di Torino, C.so Duca degli Abruzzi 24, 10129 Turin, Italy; 3 CNR-IFC, Clinical Epidemiology and Pathophysiology of Renal Diseases and Hypertension, Nefrologia-Ospedali Riuniti, 89100 Reggio Calabria, Italy; 4 S.C. Nefrologia e Dialisi, Azienda Ospedaliera di Terni, viale Tristano di Joannuccio, 05100 Terni, Italy

**Keywords:** Acute kidney injury, Artificial intelligence, eAlert, KDIGO

## Abstract

**Objectives:**

The purpose of this study was to externally validate algorithms (previously developed and trained in two United States populations) aimed at early detection of severe oliguric AKI (stage 2/3 KDIGO) in intensive care units patients.

**Methods:**

The independent cohort was composed of 10'596 patients from the university hospital ICU of Amsterdam (the “AmsterdamUMC database”) admitted to their intensive care units. In this cohort, we analysed the accuracy of algorithms based on logistic regression and deep learning methods. The accuracy of investigated algorithms had previously been tested with electronic intensive care unit (eICU) and MIMIC-III patients.

**Results:**

The deep learning model had an area under the ROC curve (AUC) of 0,907 (± 0,007SE) with a sensitivity and specificity of 80% and 89%, respectively, for identifying oliguric AKI episodes. Logistic regression models had an AUC of 0,877 (± 0,005SE) with a sensitivity and specificity of 80% and 81%, respectively. These results were comparable to those obtained in the two US populations upon which the algorithms were previously developed and trained.

**Conclusion:**

External validation on the European sample confirmed the accuracy of the algorithms, previously investigated in the US population. The models show high accuracy in both the European and the American databases even though the two cohorts differ in a range of demographic and clinical characteristics, further underlining the validity and the generalizability of the two analytical approaches.

**Graphical abstract:**

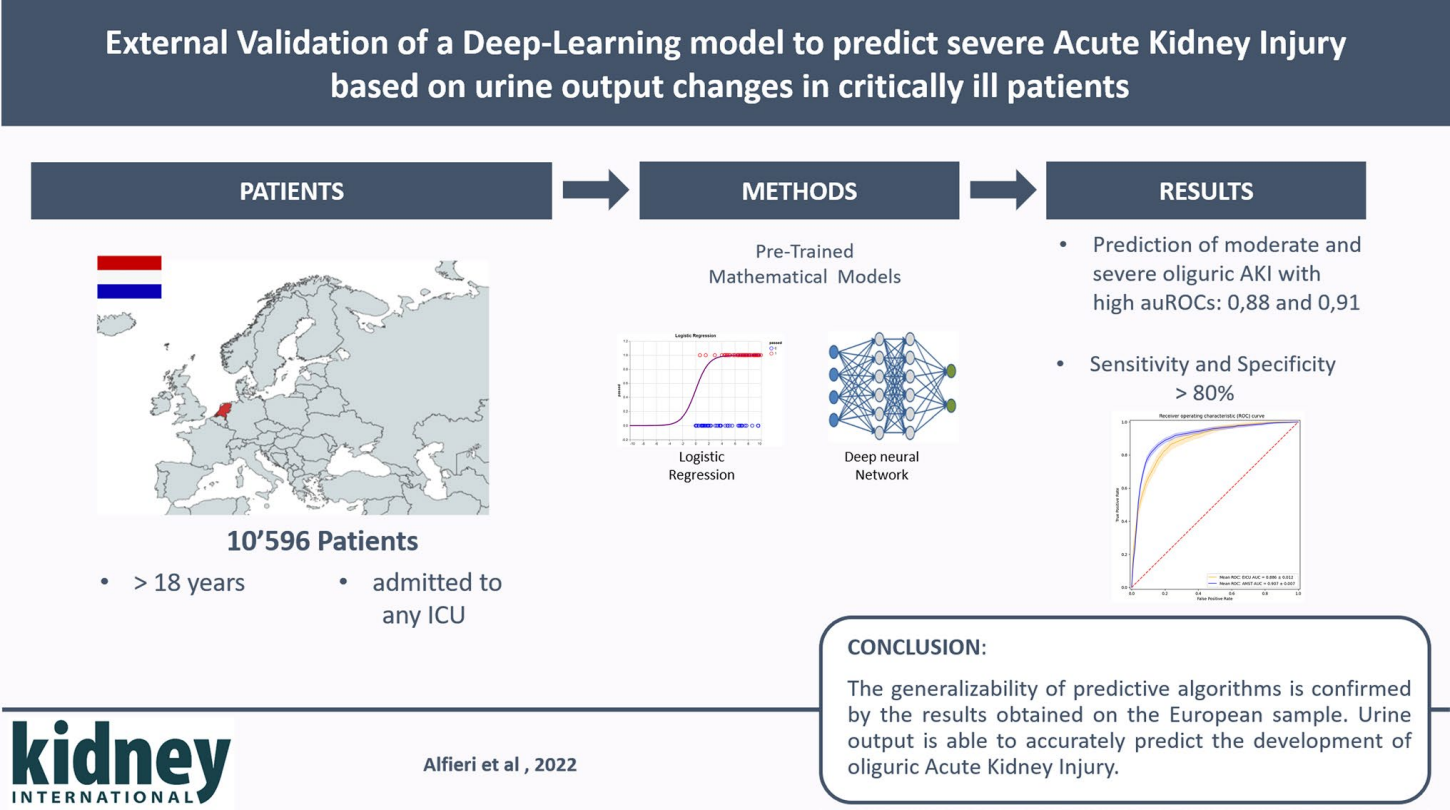

## Introduction

Acute kidney injury (AKI) is a sudden loss of excretory kidney function which represents a worldwide concern in high, middle, and low-income countries. In developed countries, AKI mostly occurs in elderly hospitalized patients and particularly in individuals admitted to intensive care units (ICUs). The clinical management of AKI in ICU patients is challenging and requires appropriate volume control, nephrotoxic drug tailoring, and the timing and type of renal replacement therapy (RRT). As AKI has a poor prognosis in ICU patients, early identification of patients at risk can be useful to reduce the adverse clinical consequences associated with AKI. Thus, early detection of the disease is essential to plan appropriate clinical surveillance in patients at risk.

As indicated in the International Society of Nephrology initiative program, the ability to predict the development of AKI through mathematical models could be an important tool to decrease preventable deaths due to AKI [1]. In our previous study [2], we focused on artificial intelligence analysis using deep-learning (DL) and logistic regression methods, and we demonstrated the accuracy of these tools to predict the development of oliguric AKI. The study was based on two clinical databases collected in the United States (US), the MIMIC-III [3] and electronic ICU (eICU) [4]. The limiting factors were the retrospective design and the need for external validation. For this reason, we conducted the same analysis on a different European database, i.e. the AmsterdamUMC [5], with the aim to externally validate the algorithms that had been previously developed and trained in the United States population, and to verify their accuracy and ability to predict the onset of AKI in hospitalized patients.

## Methods

### Data sources

The AmsterdamUMC database [5] contains anonymized data of 20'109 European patients admitted to the University hospital ICU of Amsterdam, in the Netherlands between 2003 and 2016, for a total of 23'106 admissions.

The inclusion and exclusion criteria used to determine the final patients' population information are described in our previous study [2].

The endpoint was defined as “Oliguric AKI stage 2/3” (from KDIGO classification), characterized by a simultaneous reduction in urine output and an increase in serum creatinine [6] that developed during the ICU stay. For each patient, baseline creatinine was calculated as the lowest recorded value and, in case of readmissions, the lowest available value was considered the baseline value. Urine output trend was normalized by ideal body weight (IBW). Regarding the IBW computation, in the absence of exact values of height due to the de-identifying process adopted in the AmsterdamUMC database, a mean value of the indicated height range was used.

### Data extraction and imputation of missing values

For patients who met the inclusion criteria of the study, raw data of serum creatinine values, urine output trend and demographic characteristics were extracted and processed. Urine output observations were manually reported by nurses with random sampling frequencies, whereas the creatinine values were automatically entered into the database by the laboratory when the measurement was carried out.

The data were analysed following the same procedural steps adopted in our previous study [2]. Regarding the urine output values, starting from the original recordings with an unfixed sampling frequency, we applied the following imputation technique to obtain a time series with a constant sample rate of 1 h:

- If a subject presented consecutive urine output measurements in a time interval shorter than 9 h, we calculated a cumulative total value of the measurements and then divided by the number of hours without available records and assigned it to each hour.
- If no measurement of serum creatinine was available for a time interval < 4 days, a carry-forward imputation method was employed to fill the missing hour with the last available measurement.

Missing value imputations allow to have a standardized method to provide the measurements in the algorithm.

### Mathematical models and analysis

The task of our prediction model is to predict in advance whether a patient will experience an AKI episode or not during the ICU stay. In our previous study [2], two separate machine learning techniques were compared in order to build a model able to predict the onset of hospital-acquired oliguric AKI (stage 2, 3 KDIGO).

Logistic regression is one of the simplest and most widely used models for dealing with ICU-related problems [7–9]. The input includes a series of data-dependent variables that are used to find relationships with the outcome and returns a probability of risk of being part of a class, which in our case results in the probability of the risk of developing or not oliguric-AKI 2/3 within the following 24 h.

The deep learning model is a much more complex algorithm compared to the logistic regression one. It is the evolution of artificial neural networks (ANN) where an increasing number of learning hidden layers have been included in its architecture. It is based on parallel one-dimensional (1D) convolutional layers that analyse time-series of hourly urine output in such a way to allow automatic extraction of informative characteristics from the raw data given as input and compute the probability of developing oluguric-AKI 2/3 within the following hours.

### Pre-Trained models

Logistic regression and deep learning models were pre-trained on a cohort of 21'681 patients from the MIMIC-III (10,824 patients) and eICU (10'857 patients) databases. In detail, the entire MIMIC-III database was used to train both mathematical models, while only a portion of the eICU population (44%) was employed for that purpose (the residual was split and used to validate and to test the model performances in the previous publication).

During the training phase of the algorithms, to ensure the prediction at least 6 h before the event, the urine output trend of patients with an episode of oliguric AKI 2/3 was truncated 6 h before the event; conversely, the time series of non-severe-AKI patients were used from their entry to the ICU until their discharge or death.

The same approach was applied to treat the AmsterdamUMC database patients.

The metrics that were adopted to assess the models’ predictive performances included the area under ROC curve (AuROC), sensitivity, specificity, and the positive and negative likelihood ratios.

## Results

**Fig. 1:**
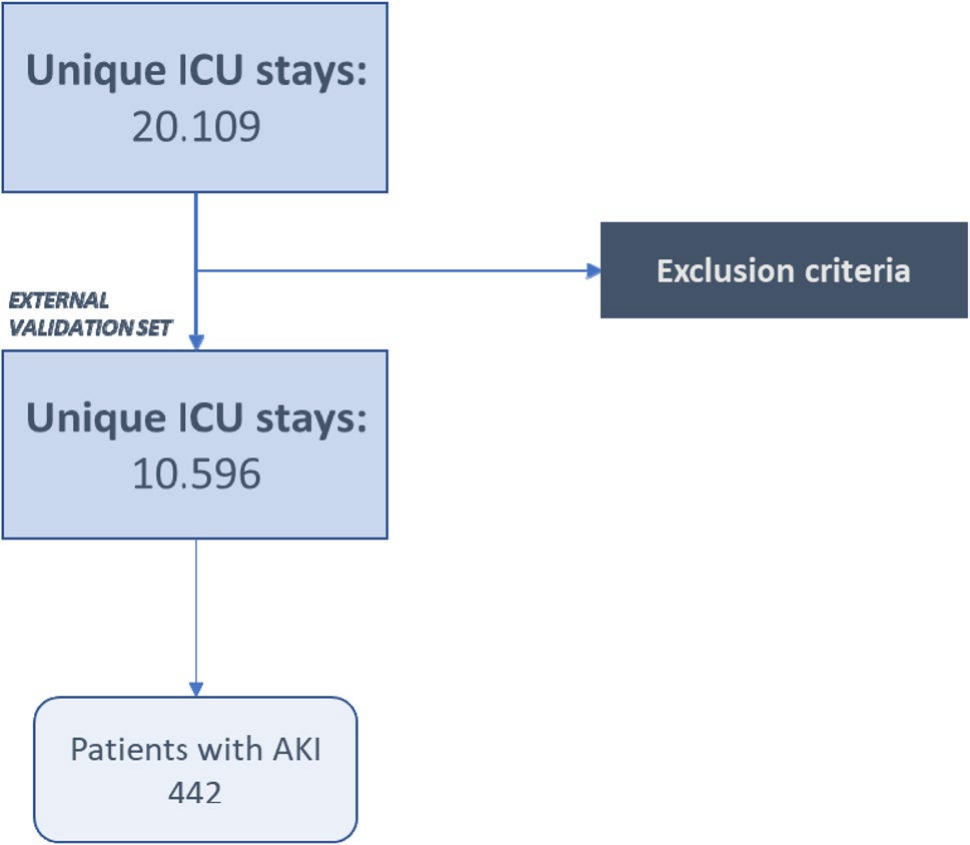
Residual ICU stays after the application of exclusion criteria

In the analysis we included the data of 10'596 patients out of the 20'109 available in the database. This is because only 52,7% of the patients met the study inclusion criteria (see Fig. 1). With regard to the exclusion criteria, incomplete or absent records of urine output were present in 17% of MIMIC III, in 64% of eICU, and in 28% of the AmsterdamUMC patients. Serum creatinine records were absent/incomplete in 4,3% of MIMIC III, in 12% of eICU, and in 34% of AmsterdamUMC patients. In the AmsterdamUMC selected population, we found that 4,2% of patients (*n* = 442) had an episode of hospital-acquired oliguric-AKI during their stay, whereas the incidence of the pathology in the eICU dataset was equal to 3%. A between-group difference was observed in the distribution of age categories above 60 years (i.e. 60–69, 70–79 and 80 + years): in the AmsterdamUMC database, the distribution of patients in these categories was 28,2%, 30,6% and 11,7%, respectively, whereas in the eICU database it was 23,3%, 23,5% and 21,7%, respectively (see Table 1). Furthermore, in the first database, we observed that 71% were male compared to 63% in the eICU dataset. Both cohorts presented the same rate (8,9%) of hospital mortality.

**Table 1:** Summary statistic of the patients’ characteristics of the AmsterdamUMC and eICU databases

	AmsterdamUMC	eICU
Patients	10'596	7'080
Oliguric-AKI 2/3	442 (4,2%)	216 (3,0%)
Gender (M)	7529 (71,1%)	4'476 (63,2%)
Age groups		
18–39	718 (6,8%)	548 (7,7%)
40–49	776 (7,3%)	526 (7,4%)
50–59	1'640 (15,5%)	1'156 (16,3%)
60–69	2'987 (28,2%)	1'652 (23,3%)
70–79	3'240 (30,6%)	1'662 (23,5%)
80 and above	1'235 (11,7%)	1'536 (21,7%)
Length of ICU stay (h)	34,6 [22,9- 87,8]	145,7 [96,7- 230,2]
In-hospital deaths	949 (8,9%)	631 (8,9%)
Min diuresis value (ml/h/kg)	0,33 [0,15- 0,56]	0,29 [0,14- 0,49]
Max diuresis value (ml/h/kg)	3,60 [2,10- 5,90]	3,95 [2,35- 6,.34]
Min serum creatinine value (mg/dL)	0,93 [0,74- 1,15]	0,86 [0,69- 1,17]
Max serum creatinine value (mg/dL)	1,02 [0,84- 1,29]	1,07 [0,84- 1,50]
Basal serum creatinine levels (mg/dL)	0,84 [0,68- 1,06]	0,8 [0,64- 1,05]

### Logistic regression model

**Fig. 2:**
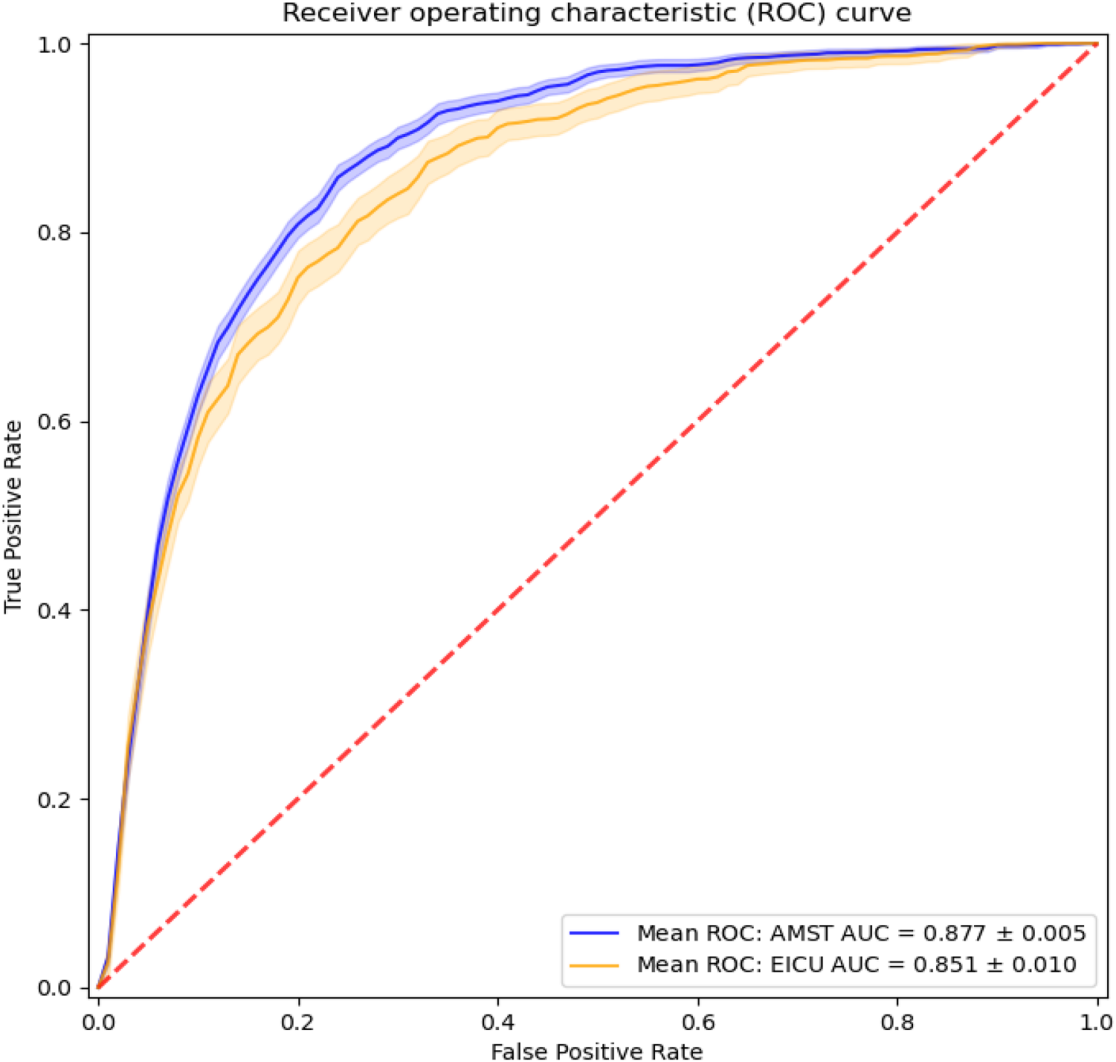
Area under the ROC (± standard error) achieved by the LR model. *AMST AUC* AmsterdamUMC database, Area Under ROC Curve, *EICU AUC* eICU database, Area Under ROC Curve

Using multi-feature logistic regression analysis, AKI prediction score showed good discriminative ability with AuROC = 0,877 (see Fig. 2). Sensitivity and specificity were 80% and 81%, respectively. The same model tested on the eICU data reported an AUC of 0,851 with 80% and 75% sensitivity and specificity, respectively (see Table 2). The positive likelihood ratio (+ LR) was 4,21 and the negative (-LR) was 0,25.

**Table 2:** Numerical results for the multi-feature logistic regression model over the two testing datasets

Model	Dataset	Working point	auROC (avg)	Sensitivity (%)	Specificity (%)	LR +	LR −
Logistic Regression	AmsterdamUMC	Sensitivity = 80%	0,88	80	81	4,21	0,25
		Knee-point		81	80	4,05	0,24
Logistic Regression	eICU	Sensitivity = 80%	0,85	80	75	3,20	0,31
		Knee-point		77	78	3,52	0,29

*auROC* area under receiving operator curve, *LR +* positive likelihood ratio, *LR −*  negative likelihood ratio

### Deep Learning model

**Fig. 3:**
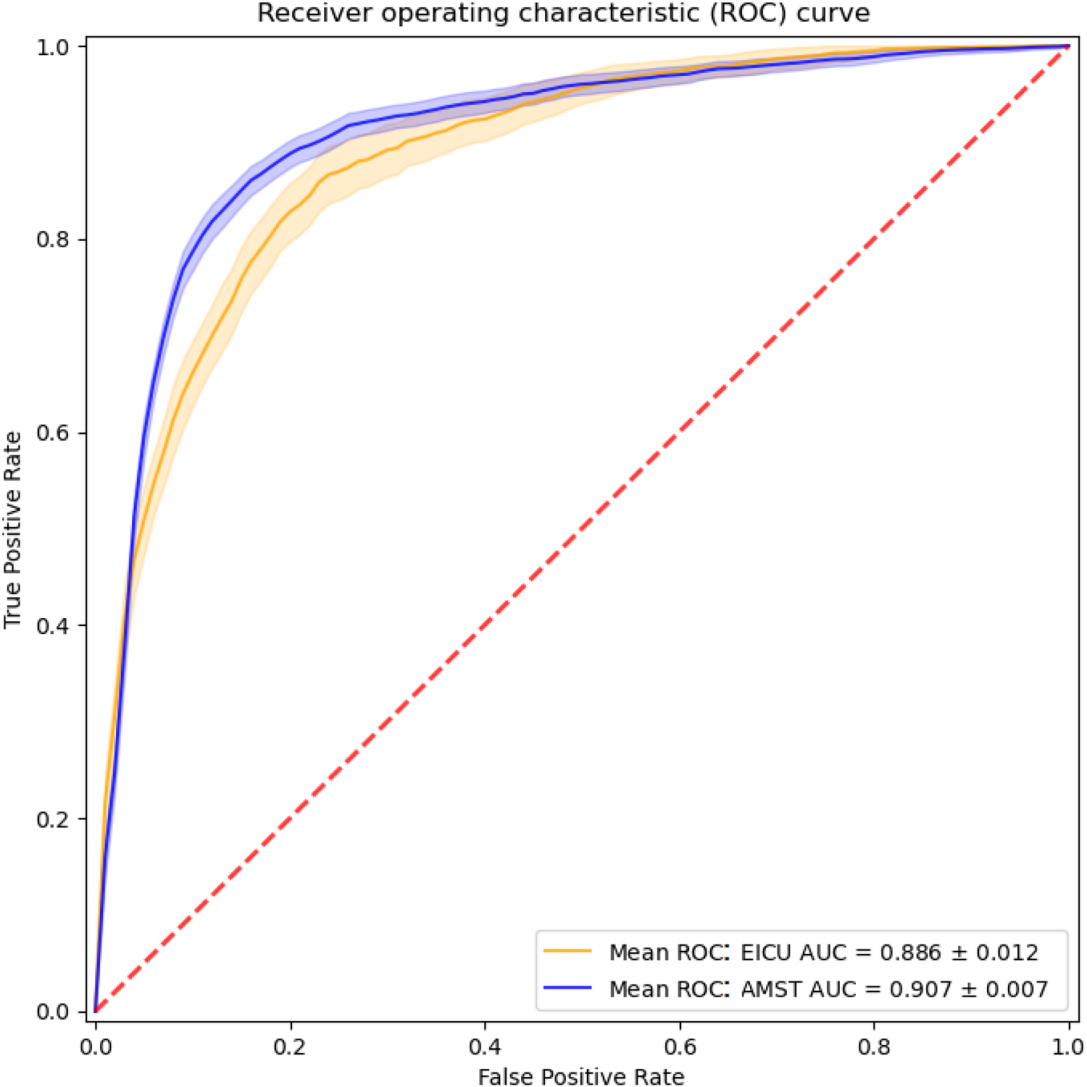
Area under the ROC (± standard error) achieved by the Deep Learning model. *AMST AUC* AmsterdamUMC database, Area Under ROC Curve, *EICU AUC* eICU database, Area Under ROC Curve

**Fig. 4:**
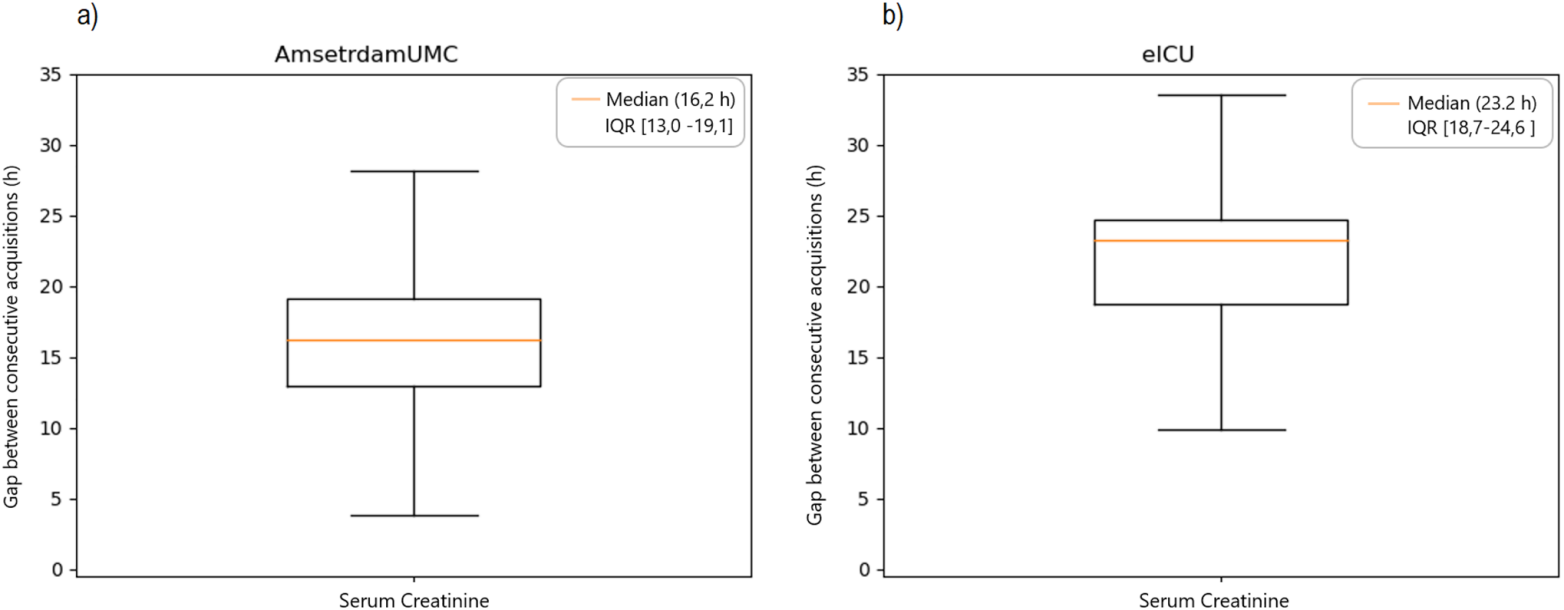
Box plots of the distributions of the real acquisition time for serum creatinine among patients of the **a** AmsterdamUMC and **b** eICU datasets

**Fig. 5:**
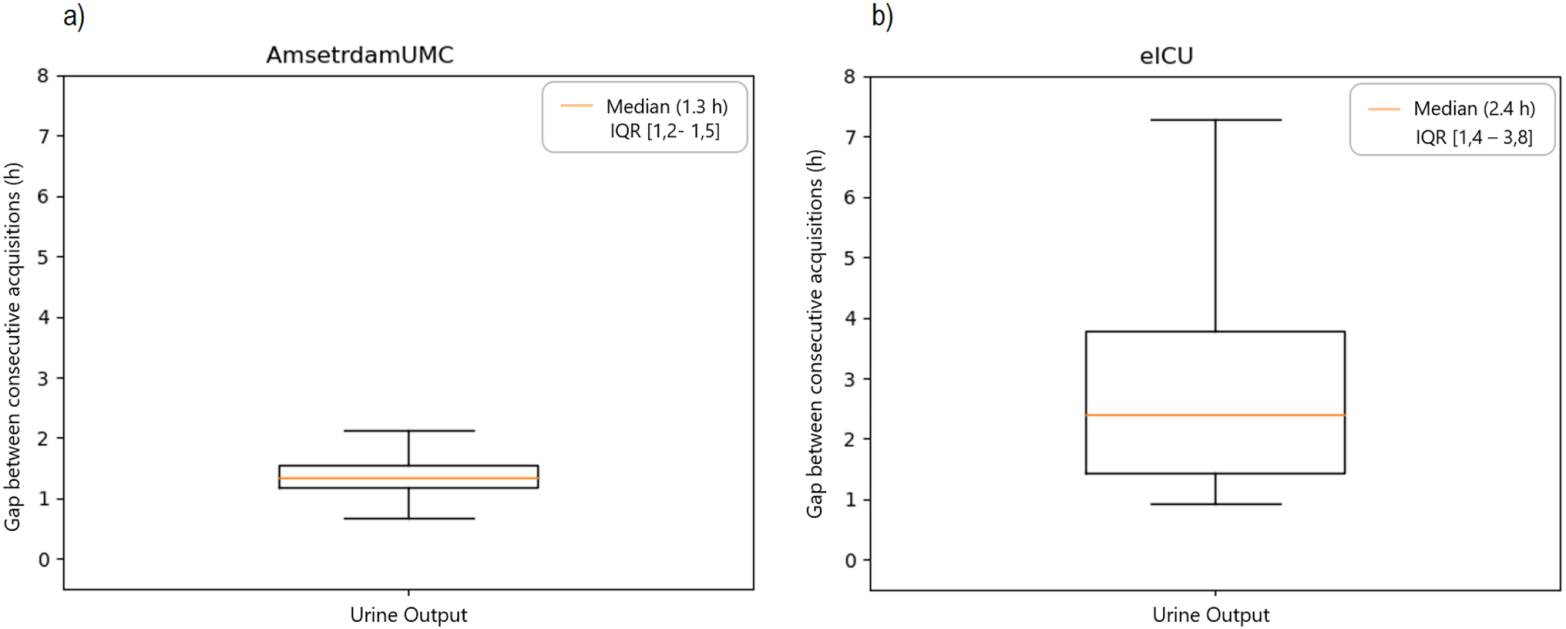
Box plots of the distributions of the real acquisition time for urine output among patients of the **a** AmsterdamUMC and **b** eICU datasets

In the Deep Learning model an AuROC of 0,907 was achieved (see Fig. 3) compared to 0,886 in the eICU, at 80% sensitivity and 89% specificity (Table 3). Albeit not significantly different, these increased performances could be attributed to the higher frequency of the data collection in the AmsterdamUMC database than in the eICU model (see Figs. 3 and 4, and Table 4). Indeed, an augmented prevalence of AKI episodes of about 4,2%, was observed compared to the incidence of 3% of AKI episodes in the USA test cohort. In the European database, the + LR was 7,27 and the -LR was 0,22. Furthermore, the AmsterdamUMC database differed from the eICU in that serum creatine and urine output were recorded more frequently (Figs. 4a, b, 5a, b, and Table 4).

**Table 3:** Numerical results for the deep learning model over the two testing datasets

Model	Dataset	Working point	auROC (avg)	Sensitivity (%)	Specificity (%)	LR +	LR −
Deep learning	AmsterdamUMC	Sensitivity = 80%	0,91	80	89	7,27	0,22
		Knee-point		85	85	5,67	0,18
Deep learning	eICU	Sensitivity = 80%	0,89	80	84	5,00	0,20
		Knee-point		82	82	4,50	0,22

*auROC* area under receiving operator curve, *LR +* positive likelihood ratio, *LR −* negative likelihood ratio

**Table 4:** Median acquisition frequency and interquartile range of urine output and creatinine for patients of the AmsterdamUMC and eICU datasets

	eICU	AmsterdamUMC	*p* value
Patients	7'080	10'596	
Median acquisition frequency of creatinine	23,2 h [18,7–24,6]	16,2 h [13,0–19,1]	< 0,0001
Median acquisition frequency of urine output	2,4 h [1,4–3,8]	1,3 h [1,2–1,5]	< 0,0001

## Discussion

In our previously published study, we investigated the accuracy of a deep-learning model based on urine output to predict oliguric AKI in the ICU [2]. In that study we demonstrated that the analysis of 12-h urine output with a deep learning model had good diagnostic performance, with an area under Receiving Operator Curve of 0,89 ± 0,01 (sensitivity 80% and specificity 84%). The estimated ability of the model to predict AKI stage 2 and 3 was of at least 12 h before the development of the event, with a + LR of 4,87 and 5,06 and a − LR of 0,24 and 0,20 respectively. In that study we used the data of the clinical databases collected in the US, the MIMIC-III and eICU. The use of retrospective data represents the main limitation of our investigation and, furthermore, the results need to be externally validated.

For this reason, in the current study we conducted the same analysis on a different database with the scope of externally validating the algorithms that had previously been developed and trained in the US population. The “AmsterdamUMC database” contains data concerning 23'106 admissions to the ICU of the University of Amsterdam observed and recorded between 2003 and 2016 [5]. By comparing the analysed data with the eICU results, we observed a higher auROC (0,907 ± 0,007), a better + LR of 7,27 and a − LR of 0,22 for the AmsterdamUMC. This analysis allowed us to confirm the previous results with an external validation. Furthermore, we observed a slightly better performance of the deep learning model in the European dataset. Indeed, we found that the two databases have some differences and this may have led to a higher auROC and + LR. Particularly, the European database presented a higher incidence of AKI stage 2 and 3 and higher frequency of serum creatinine and urine output measurements.

The AmsterdamUMC database reported an AKI incidence of 4,2% vs 3% in the eICU: the most highly represented age group was 70–79 yrs (355%) in the AmsterdamUMC compared to 60–69 years (26,8%) in the eICU.

Serum creatinine was measured with a median acquisition frequency of 16,2 h instead of 23,2, and, more remarkably, the acquisition frequency of urine output in AmsterdamUMC was 1,3 h compared to 2,4 in the eICU (median values). Concerning hourly detection of urine output and serum creatinine monitoring, the percentage of database patients with a decreased frequency of observation is important. This fact represents the necessity to improve patient monitoring at least in established specific realities or situations. Nevertheless, considering the patients included in data analysis, it appears that the AmsterdamUMC database has a greater  precision of variable acquisition and a relatively slightly higher number of events.

The possible connection between the higher frequency of observations and events and the observed differences on the positive likelihood ratio was not tested in our study. However, some issues may arise from our observations. The first aspect is the reason for investigating urine output in terms of a predictive outcome. It is known that AKI can develop in two different forms, i.e., oliguric (according to the KDIGO classification) and non-oliguric disease. Oliguric AKI represents about 40% of the disease, with a relevantly higher mortality (50–60%), both in the ICU and in hospital settings, than the non-oliguric form (10–20%) [10, 11]. The second issue, which is strictly connected to the first, is the definition of oliguria. The KDIGO definition of oliguria considers a urine output criteria of < 0,5 ml/kg/h for a period of 6–12 h (AKI stage 1) or > 12 h (stage 2), and < 0,3 ml/kg/h for a period ≥ 24 h or anuria for a time ≥ 12 h, taking into account “consecutive” hours (stage 3). However, this classification is under debate because the incidence of AKI and related stage can differ when considering an average value of urine output for a determined period of time or a series of consecutive values [12]. Finally, the third aspect is the inverted relationship between the predictivity of the urine output and the serum creatinine levels. Vincent et al. observed that oliguria on admission is present in about 25% of ICU patients and the mortality rate is twice as high compared to non-oliguric patients [13]. They also reported differences in mortality depending on the ability to restore normal urine output within the first 48 h of ICU stay: the mortality rate is no different from the values observed in non-oliguric patients. They defined these patients as having “transient oliguria” and they made up 30% of the oliguric patients at ICU admission. The authors observed that oliguria averaged over 6 h had a greater sensitivity to predict AKI stage 1. Furthermore, the predictivity of oliguria on dramatic events, such as AKI development or hospital mortality, could be linked not only to the time window (how many hours) but also to the volume  of diuresis, as demonstrated by the study of Ralib et al. [14]. In their study the current AKI definition was based on a *“too liberal” urine output*, because the authors observed that *“a 6-h urine output threshold of 0,3 ml/kg/hour best associated with mortality and dialysis”*.

Regarding the accuracy of a predictive model with reference to the possible occurrence of a given event, the analysis of the ROC curve is commonly used. This analytical approach provides a graph that defines the sensitivity (i.e. the true positive rate) versus 1—specificity (i.e. the false-positive rate) for each possible cut-off of the prediction rule. In our analysis, we found areas under ROC curves to be consistently high (> 85%) in both cohorts and when using the analysis of both logistic regression or deep learning models (Tables 2, 3). We defined the results also in terms of positive and negative likelihood ratios, which represent the indexes that combine sensitivity and specificity. The + LR relates to a positive diagnosis in patients with a positive test and it is calculated as sensitivity/(1-specificity), with a value > 1. The + LR values that were obained by logistic regression were 4,21 (at a fixed sensitivity of 80%) and 4,05 (knee-point) in the Amsterdam cohort, whereas they were 3,20 (at a fixed sensitivity of 80%) and 3,52 (knee-point) in the eICU cohort (see Table 2). Of note, higher + LR values were observed in both cohorts when the deep learning model was applied in the analysis (Table 3). In particular, in the Amsterdam cohort the + LR of 7,27 (at a fixed sensitivity of 80%, Table 3) indicated a sevenfold increase in terms of odds of having the event in a patient with a positive test result. Therefore, the higher the level of + LR, the more informative the test.

The − LR gives the indication of having a diagnosis in patients with a negative test. It is calculated as (1—sensitivity)/(specificity) and its value is usually < 1. We found a − LR of 0,22 in the Amsterdam cohort (at a fixed sensitivity of 80%, Table 3) that indicated a 4,5-fold decrease in terms of odds of having the event of interest in a patient with a negative test result. Consequently, the smaller the − LR value, the more informative the test.

To date, there is scarce evidence in the literature concerning the accuracy of a predictive model in the urine output and AKI events. Macedo et al. reported a + LR of 1,25 and a − LR of 0,92 for 12-h oliguria in AKI stage 2, whereas the levels reported for 24 h oliguria in AKI stage 3 were 2,0 and 0,96, respectively [15]. Another study involving cardiac surgery ICU patients indicated a + LR of 2,9 and a − LR of 0,45 in case of 6-h oliguria in AKI stage 1 [12]. Based on this literature, it is important to point out that the accuracy of our prediction models is consistently higher than those provided by previous prediction rules and is in accordance with our previous study [2]. A different approach to AKI prediction is the use of furosemide as a bolus and the consequent monitoring of diuresis for a 2-h period, i.e., the furosemide stress test (FST) [16]. The test investigates the integrity of tubular function and predicts the worsening from AKI stage 1 or 2 to AKI stage 3 or the need for dialysis. The predictive capacity is high, as represented by the AUC equal to 0,87, with a sensitivity of 87,1% and a specificity of 84,1%. An increase in predictivity was obtained by coupling FST with biomarkers [17]. In patients with urine TIMP-2xIGFBP-7 > 0,3, the AUC for AKIN stage 3 progression increases up to 0,9, and for RRT it increases up to 0,91. Although FST is a reliable tool for the prediction of AKI worsening, as both the meta-analysis by Chen [18] and the review by Coca [19] highlight, debate still exists on the heterogeneity of the studies, the number of enrolled patients, the type of study design, the severity of basal AKI, the role of albumin levels on furosemide sensitivity, the ability of continuous furosemide infusion to increase sensitivity and specificity of the test, as Mariano suggests [20, 21], and the ability of AUC values to define the predictive capacity of a test.

Nevertheless, the application of a machine learning method differs from FST in several aspects.

The first is the target population: we applied the predictive model to all patients admitted to the ICU, with the exclusion of patients in need of continuous RRT (CRRT) during the stay, and of those with community-acquired AKI. We obtained excellent results in terms of AUC, positive and negative likelihood ratio when oliguric AKI was considered. On the contrary, the study by Chawla and Koiner applied FST to selected patients with AKIN stage 1 and 2, presence of granular or epithelial cell casts on urine sediment, or FeNa > 1%. In both studies patients were well resuscitated, sufficiently clinically stable and euvolemic.

The second difference is the moment of AKI prediction and its severity: FST is applied when a patient has AKIN stage 1 and 2 to predict AKI stage 3, including the need for hemodialysis. Our study continuously analyses the urine output in patients without AKI or with AKI stage 1 to predict AKI stage 2 and stage 3, with the exclusion of patients who require dialysis. Patients were included regardless of therapeutic intervention or volume cut-off, during all the ICU stays and by adopting 12-h periods of observation in sliding windows, to predict AKI stage 2 or 3.

The artificial intelligence that analysed the data, which included urine production to predict AKI events by exploiting the deep learning model, seems to overcome the KDIGO classification of urine output in terms of quantity and time. Actually, the KDIGO classification presented low accuracy in predicting higher stages of AKI development. In our study we have tested a mathematical model, derived from Artificial Intelligence process, which has a high accuracy to predict AKI stage 2/3 future events. In our previous analysis, the highest observed + LR was 5,00 and the lowest –LR was 0,20. As mentioned above, in the present study we used a different database with a different approach towards urine output analysis, and an artificial intelligence application characterized by the deep learning process. Furthermore, by comparing the results with the current study, a + LR equal to 5,00 demonstrated a moderate increase in the probability of a disease, given by a positive test.

This study also presents some limitations regarding the design since it relies on a retrospective source of data. Indeed, we documented an important difference between the two databases in terms of frequency of data acquisition that can significantly affect the validity of the analysis. The error in the manual determination of urine output can be estimated at around 20–26% [21]. Another limiting factor is the usefulness of the information derived from DL analysis on everyday clinical work. For this reason, it appears necessary to develop an observational study in which a precise method of urine output recording is well established. Based on this consideration, we have already designed an observational prospective study which should start soon: all institutions interested in participating can contact our research group for further information and centre enrolment.

Finally, an interventional study designed to compare the use of electonic alarm is needed. It should be based on a highly accurate prediction model, as the one we have provided here, based on the routine clinical procedures of ICU mortality, length of stay or AKI development.

## Conclusions

In conclusion, through external validation on a European sample, we confirmed the accuracy of the algorithms that we previously investigated in the US cohort. It is also important to underline that, although the two study cohorts differ with regard to some demographic and clinical data, we observed similar accuracy in both regression models (Logistic Regression and Deep Learning). This evidence supports the validity and the generalizability of the two analytical approaches. Precise monitoring of urine output can be obtained by an automatic device, which could be interfaced with a predictive algorithm to generate a warning for the possible development of AKI stage 2 or 3. In terms of care opportunity, increased attention by the medical staff could lead to the diagnosis of an evolving clinical condition and thus to the early use of “precision therapy”, in an effort to decrease in-hospital mortality, kidney damage, disability and length of hospital stay.
